# The role of leptin in regulation of the soluble amyloid precursor protein α (sAPPα) levels in lung cancer cell media

**DOI:** 10.1038/s41598-024-55717-y

**Published:** 2024-02-28

**Authors:** Ben Haddad, Jeneen Khalil, Hind Al Khashali, Ravel Ray, Stuti Goel, Ban Darweesh, Kai-ling Coleman, Caroline Wozniak, Robert Ranzenberger, Brooke Lopo, Jeffrey Guthrie, Deborah Heyl, Hedeel Guy Evans

**Affiliations:** Chemistry Department, Eastern Michigan University, Ypsilanti, MI 48197 USA

**Keywords:** Biochemistry, Cell biology

## Abstract

Previously, we found that the levels of soluble amyloid precursor protein α (sAPPα) are regulated, in part, by acetylcholinesterase (AChE) in human A549 (p53 wild-type) and H1299 (p53-null) NSCLC cell lines. In this study, we found regulation of sAPPα levels in the media by leptin, a widely recognized obesity-associated adipokine that has recently been shown to play a possible role in cancer signaling. Increased levels of sAPPα, that were accompanied by lower Aβ40/42 levels in the media of A549 and H1299 cells, were detected upon cell incubation with leptin. Conversely, knockdown of leptin or its receptor led to reduced levels of sAPPα and increased levels of Aβ40/42 in the media of A549 and H1299 cells, suggesting that leptin likely shifts APP processing toward the non-amyloidogenic pathway. A549 cell treatment with leptin increased acetylcholine levels and blocked the activities of AChE and p53. Treatment with leptin resulted in increased activation of PKC, ERK1/2, PI3K, and the levels of sAPPα, effects that were reversed by treatment with kinase inhibitors and/or upon addition of AChE to A549 and H1299 cell media. Cell viability increased by treatment of A549 and H1299 cells with leptin and decreased upon co-treatment with AChE and/or inhibitors targeting PKC, ERK1/2, and PI3K. This study is significant as it provides evidence for a likely carcinogenic role of leptin in NSCLC cells via upregulation of sAPPα levels in the media, and highlights the importance of targeting leptin as a potential therapeutic strategy for NSCLC treatment.

## Introduction

Worldwide, lung cancer remains widespread and a leading cause of mortality with non-small cell lung cancer (NSCLC) accounting for ~ 80% of all lung cancer cases^1,2^. Compared to small-cell carcinoma, NSCLCs are relatively insensitive to chemotherapy^2–4^.

Cancer and Alzheimer’s Disease (AD) are among the most intensely investigated age-related diseases^5–12^. Several studies have demonstrated an inverse relationship between the incidence and prevalence of cancer and AD in that cancer pathogenesis appears to protect patients against AD and vice versa^5,8,10–12^. Signaling cascades and molecular mechanisms related to cell growth and proliferation are reported to be decreased in AD and increased in cancer^8,9,11,12^.

Among several identified molecular players that have cancer-promoting and AD-inhibiting properties is leptin^12–14^. Leptin is known to be a pleiotropic hormone secreted primarily by adipocytes regulating energy metabolism^12,15–17^. More recently, however, leptin was shown to be synthesized and secreted by several non-adipose tissues including lung tissue, and both leptin and its receptors are considered to play a role in the development and progression of a variety of tumors^15,17^. The leptin receptor, a type 1 cytokine receptor, has been shown to be highly abundant in a broad range of cancer types with an important role in the escalation and pathogenesis of many tumors via activation of signaling cascades crucial for cancer cell growth^16–23^. Leptin was shown to be upregulated in lung tumors compared with normal lung increasing their growth, proliferation, and metastasis, with anti-apoptotic functions^15,17,20,21^. A positive correlation was observed with increased malignancy of different cancer subtypes and leptin expression^15–17,20,24^.

Leptin has been found to reduce the levels of toxic extracellular amyloid beta (Aβ) and induce protective functions in a range of neurodegenerative models^14^. Amyloid precursor protein (APP) is a type 1 transmembrane glycoprotein with a central role in AD, and neuronal homeostasis^25–27^. Several reports have shown ubiquitous expression of APP by both neuronal and non-neuronal cells and frequent overexpression in a number of cancers including lung cancer, increasing cell growth and proliferation^25,28^. APP can be processed into biologically active fragments via sequential site-specific proteolytic cleavages through an amyloidogenic or a non-amyloidogenic pathway^26,29^. In the amyloidogenic pathway, APP is sequentially cleaved by β-secretase and γ-secretase, generating Aβ40 and Aβ42^26,29,30^. Several malignancies have been associated with the non-amyloidogenic pathway where APP is cleaved within the Aβ sequence at the plasma membrane by α-secretase, mainly carried out by the disintegrin and metalloproteinase domain protein (ADAM) family, generating soluble amyloid precursor protein α (sAPPα) known to be non-amyloidogenic and a growth factor for epithelial tissue^30^. The protective functions of leptin were found to be in part attributed to promoting cleavage of the transmembrane APP by α-secretase^14^.

Previously, we found that the humanin peptide blocks the aggregation of Aβ induced by acetylcholinesterase (AChE)^31^ and that the interaction of Aβ with humanin and AChE is modulated by ATP^32^. Using human A549 (p53 wild-type) and H1299 (p53-null) NSCLC cell lines^33,34^, we have previously found higher intact levels of Aβ40/42 in the media of A549 than H1299 cells that was likely due, in part, to increased proteolysis of Aβ40/42 by the matrix metalloproteinase, MMP2, in H1299 cell media^35^. We also reported minimal levels of AChE in H1299 cell media as compared to the media of A549 cells^36^ and higher levels of mature brain-derived neurotrophic factor in the media of H1299 cells than in A549 cell media^37^. More recently, we showed that sAPPα levels are regulated by AChE and mBDNF and are higher in the media of H1299 cells compared to A549 cell media^38^.

Results from this study are significant because we uncovered a role of leptin as a molecular player involved in regulation of sAPPα levels in the media of NSCLC cells through down regulation of p53 and AChE and upregulation of PKC, ERK1/2, and PI3K signaling pathways. These findings shed light on the role leptin plays in the survival of NSCLC cells and highlight a potential therapeutic approach (Supplementary Figures).

## Results

### Incubation with leptin increased the levels of sAPPα in the media of A549 and H1299 cells

**Figure 1:**
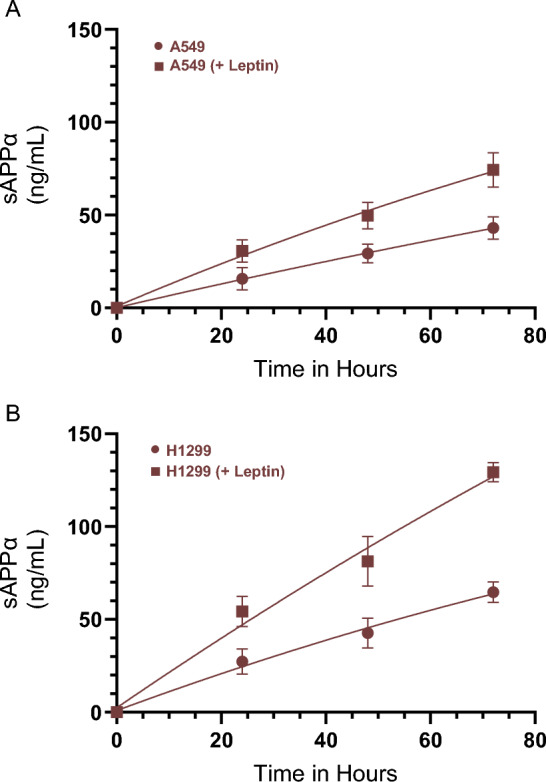
Treatment of A549 and H1299 cells with leptin increased the levels of sAPPα in the media. Cells (0.2 × 10^5^) were grown in 10% FBS-supplemented media for 24 h. The following day, the cell monolayers were incubated in serum-free media for 24 h, then the media was replaced with fresh serum-free media (0 h). The cells (**A**,**B**) were then incubated for 72 h without or with leptin (50 ng/mL). The same amount of protein (3 µL of 600 µg/mL total protein) of the media was used to quantitate the levels of sAPPα as a function of time (“Methods”). Data from five independent assays, each carried out in triplicate, were averaged. The data summarize the results expressed as means ± SD (n = 5) using the GraphPad 10.0.2 software.

Using A549 and H1299 NSCLC cells, we have previously reported that the levels of sAPPα were higher in the media of H1299 cells compared to A549 cell media^38^. Previous reports showed that leptin reduced the levels of toxic extracellular Aβ, promoted cleavage of the transmembrane APP by α-secretase, inducing protective functions in a range of neurodegenerative models^14^. To test the effects of leptin, if any, on the levels of sAPPα in A549 and H1299 cell media, cells were grown in 10% FBS-supplemented media for 24 h, then serum starved overnight. The cells were then incubated for 72 h with or without leptin (Fig. 1), then the levels of sAPPα were quantitated (“Methods”). Consistent with our previous findings, the levels of sAPPα were ~ 1.5-fold higher in the media of H1299 cells compared to A549 cell media (Fig. 1)^38^. Incubation of A549 cells with leptin increased the levels of sAPPα in the media ~ 1.70-fold after 72 h incubation (Fig. 1A) while ~ 2.0-fold increase in sAPPα levels were observed in H1299 cell media under the same conditions (Fig. 1B).

### Higher levels of sAPPα were accompanied by lower Aβ40/42 levels in the media of A549 and H1299 cells upon cell treatment with leptin

**Figure 2:**
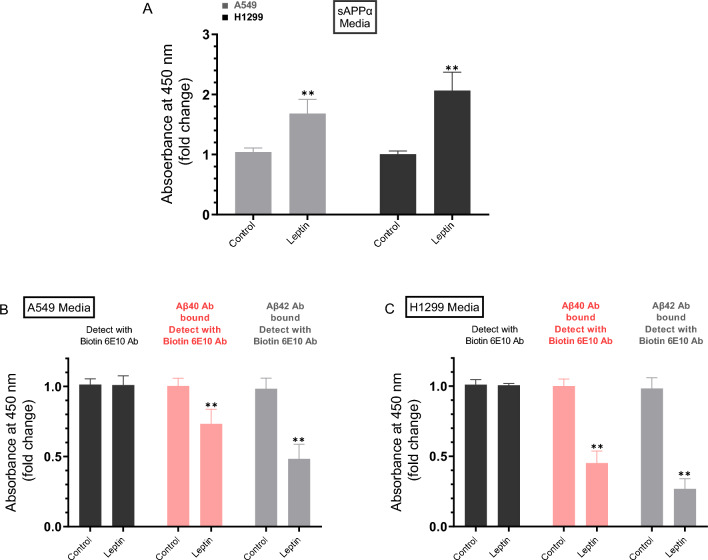
Treatment with leptin led to opposite effects on the levels of sAPPα and Aβ40/42 in the media of A549 and H1299 cells. Cells (0.2 × 10^5^) were grown in 10% FBS-supplemented media for 24 h. The following day, the cell monolayers were incubated in serum-free media for 24 h, then incubated for 72 h without or with leptin (50 ng/mL). The media was then collected and the same amount of protein (3 µL of 600 µg/mL total protein) of each sample was used to quantitate Aβ and sAPPα (**A**–**C**, “Methods”) using the indicated antibodies. Data from five independent assays, each carried out in triplicate, were averaged, normalized, and expressed as fold change relative to untreated controls using the GraphPad 10.0.2 software. The graphs summarize the results expressed as means ± SD (n = 5). Asterisks indicate a statistically significant difference from the corresponding control of each cell line. Absence of asterisks indicates no significance, Mann–Whitney test, **p < 0.01.

Leptin is known to exhibit protective actions in a number of neurodegenerative models, in part by increasing cleavage of APP by α-secretase, leading to decreased toxic Aβ levels^14^. Using A549 and H1299 NSCLC cells, we have previously reported higher intact levels of Aβ40/42 in the media of A549 cells than in H1299 cell media^35^ that correlated with higher sAPPα levels in the media of H1299 cells compared to A549 cell media^38^. To examine how leptin might affect the levels of sAPPα and Aβ40/42, cells were grown in 10% FBS-supplemented media for 24 h then serum starved overnight. The cell monolayers were then incubated for 72 h with or without leptin (Fig. 2). The levels of Aβ and sAPPα in the media were then quantitated (“Methods”).

Treatment of A549 cells with leptin increased the levels of sAPPα in the media 1.70-fold while that increase was ~ 2.00-fold in H1299 cell media (Fig. 2A). Detection using only the biotin 6E10 antibody showed no difference in the signal in the media of either A549 cells (Fig. 2B) or H1299 cells (Fig. 2C) treated with leptin. The signal detected by the 6E10 antibody is likely due to Aβ and sAPPα since this antibody is known to recognize an epitope in the first 16 amino acids of the Aβ domain, also present in sAPPα, but absent in sAPPβ^39–41^.

Treatment of A549 cells with leptin resulted in ~ 1.35-fold and ~ 2.00-fold decrease in the levels of Aβ40 and Aβ42, respectively (Fig. 2B) while leptin treatment of H1299 cells led to ~ 2.20-fold decrease in the levels of Aβ40 and ~ 3.70-fold decrease in Aβ42 levels in the media (Fig. 2C).

### Knockdown of leptin or its receptor led to decreased levels of sAPPα and increased levels of Aβ40 and Aβ42 in the media of A549 and H1299 cells

**Figure 3:**
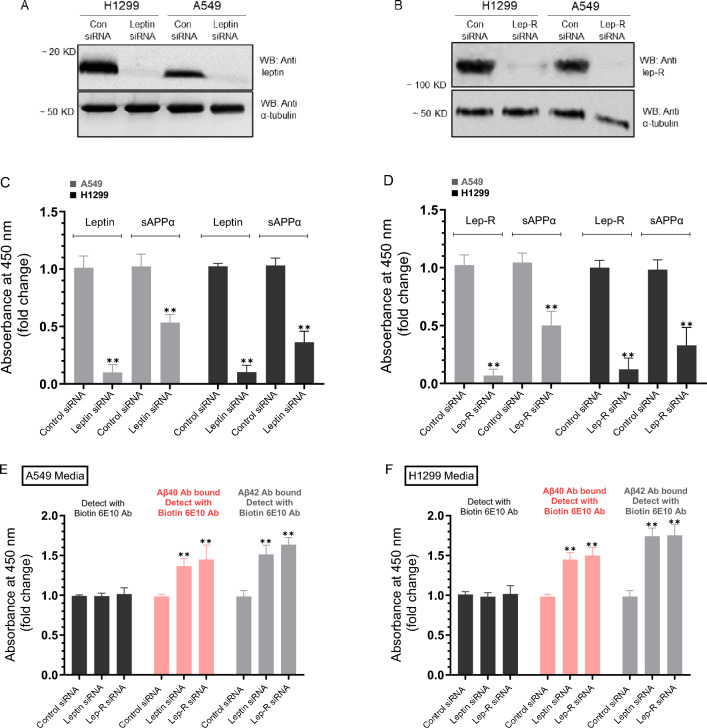
The levels of sAPPα decreased in the media of A549 and H1299 cells upon knockdown of leptin or its receptor (Lep-R) while the converse was observed for the levels of Aβ40 and Aβ42 under the same conditions. Cells (0.2 × 10^5^) were grown in 10% FBS-supplemented media for 24 h. The following day, the cell monolayers were incubated in serum-free media for 24 h, then the media was replaced with fresh serum-free media. The cells were then treated for 72 h with the indicated siRNAs as described in the “Methods” section. (**A**,**B**) Western blots of cell transfections using the indicated siRNAs and antibodies targeted against leptin, Lep-R, or α-tubulin. Leptin (**C**) or Lep-R (**D**) were quantitated as described in the “Methods” section. The media was collected and the same amount of protein (3 µL of 600 µg/mL total protein) of each sample was used to quantitate Aβ and sAPPα (“Methods”) using the indicated antibodies (**C**–**F**). Data from five independent assays, each carried out in triplicate, were averaged, normalized, and expressed as fold change relative to control siRNA using the GraphPad 10.0.2 software. The data summarize the results expressed as means ± SD (n = 5). Asterisks indicate a statistically significant difference, Mann–Whitney test from the corresponding controls of each cell line, **p < 0.01.

Our data (Fig. 2) show that treatment with leptin resulted in opposite effects on the levels of sAPPα and Aβ40/42 in the media of A549 and H1299 cells. To investigate whether knockdown of leptin or its receptor (Lep-R) had similar effects, cells were grown in 10% FBS-supplemented media for 24 h, serum-starved overnight, then the media was replaced with fresh serum-free media. The cells were then treated for 72 h with the indicated siRNAs (Fig. 3).

Knockdown of leptin or its receptor was effective in both A549 and H1299 cells (Fig. 3A,B). Compared to cells treated with control siRNA, the levels of sAPPα decreased ~ 1.85-fold and ~ 2.75-fold in A549 and H1299 cells transfected with leptin siRNA, respectively, (Fig. 3C). Comparable results were obtained upon knockdown of Lep-R in both cell lines (Fig. 3D). These results correlated with ~ 1.35-fold increase in the levels of Aβ40 and ~ 1.50-fold increase in the levels of Aβ42 in the media of A549 cells transfected with siRNA targeted against either leptin or Lep-R compared to cells transfected with control siRNA (Fig. 3E). Similarly, transfection using leptin or Lep-R siRNA increased the levels of Aβ40 ~ 1.45-fold and Aβ42 ~ 1.75-fold in the media of H1299 cells (Fig. 3F). Detection using only the biotin 6E10 antibody showed no difference in the signal in the media of either A549 cells (Fig. 3E) or H1299 cells (Fig. 3F) treated with leptin or Lep-R siRNA compared to cells transfected with control siRNA. The signal obtained upon using the 6E10 antibody likely represents both Aβ and sAPPα since 6E10 detects an epitope in the first 16 amino acids of the Aβ domain, also present in sAPPα, but absent in sAPPβ^39–41^. The results obtained upon knockdown of leptin, or its receptor (Fig. 3) are opposite to those obtained upon incubation with leptin (Fig. 2).

### A549 cell treatment with leptin increased ACh levels and blocked the activities of p53 and AChE while opposite effects were observed upon knockdown of either leptin or its receptor

**Figure 4:**
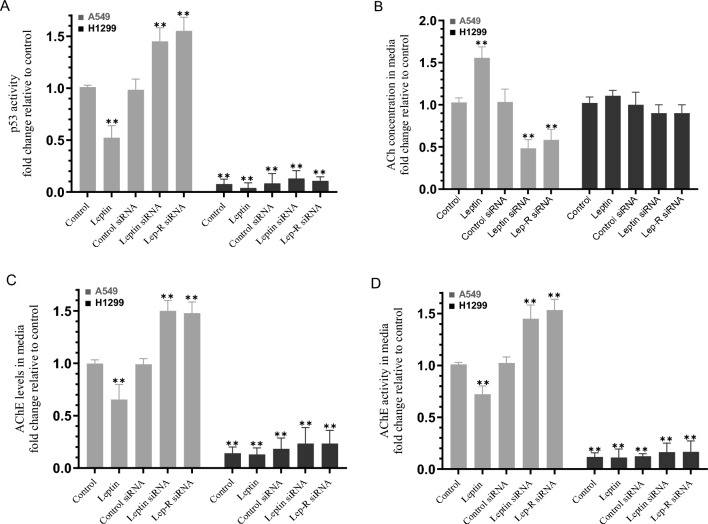
Treatment of A549 cells with leptin increased ACh levels and inhibited the activities of p53 and AChE while opposite effects were found upon transfection with siRNA targeting either leptin or Lep-R. Cells (0.2 × 10^5^) were grown in 10% FBS-supplemented media for 24 h. The following day, the cell monolayers were incubated in serum-free media for 24 h, then the media was replaced with fresh serum-free media. The cells were then either untreated or treated as indicated with leptin (50 ng/mL) or the indicated siRNAs and allowed to incubate for 72 h. The activity of p53 (**A**) in cell lysates, the levels of ACh in the cell media (**B**), the levels of AChE (**C**) and activity (**D**) in the media were measured as described in the “Methods” section. The same amount of protein (3 µL of 600 µg/mL total protein) was used for all assays. The graphs summarize the results expressed as means ± SD (n = 5) using the GraphPad 10.0.2 software. Fold change was calculated relative to the control of each cell line (**B**) or to the A549 control (**A**,**C**,**D**). Asterisks indicate a statistically significant difference from the corresponding control, Mann–Whitney test. Statistical differences between different groups were analyzed by an ordinary one-way analysis of variance (ANOVA) followed by Tukey’s post-hoc multiple comparison test, **p < 0.01. Absence of asterisks indicates no significance.

Previously, higher expression of the leptin gene was reported to negatively affect p53 signaling in NSCLC^42^. We, therefore, tested the effect of cell treatment with leptin or knockdown of either leptin or Lep-R on the activity of p53 under our conditions. Cells were grown in 10% FBS-supplemented media for 24 h, then incubated in serum-free media overnight. The media was next replaced with fresh serum-free media and the cells were then treated as indicated for 72 h with leptin (50 ng/mL) or the indicated siRNA (Fig. 4). The p53 activity decreased ~ 2.00-fold in A549 cells treated with leptin while relative to control siRNA treatment, the activity of p53 increased ~ 1.50-fold upon transfection with siRNA targeting either leptin or its receptor (Fig. 4A). No effects were observed when using H1299 cells which is an expected result since H1299 cells are known to be p53-null (Fig. 4A)^33,34^. Previously we reported that insulin-like growth factor-binding protein 3 (IGFBP-3) inhibits hyaluronan-CD44 signaling via a mechanism that depends on both p53 and AChE and that treatment of A549 cells, transfected with either p53 siRNA or AChE siRNA, with IGFBP-3 resulted in decreased AChE levels and activity in the media^36^. We also reported lower levels of AChE in the media of H1299 cells as compared to A549 cell media^36,38^. Treatment of A549 cells with leptin resulted in ~ 1.55-fold increase in the levels of ACh while no significant differences were observed in those levels in H1299 cells media (Fig. 4B). Transfection of A549 cells with siRNA targeting either leptin or its receptor resulted in ~ 2.00-fold decrease in the levels of ACh (Fig. 4B). These results correlated with ~ 1.50-fold decrease in AChE levels (Fig. 4C) and ~ 1.40-fold decrease in the activity of AChE (Fig. 4D) in the media of A549 cells treated with leptin and, conversely, ~ 1.50-fold increase in the levels and activity of AChE in the media of A549 cells transfected with siRNA targeting either leptin or its receptor compared to control siRNA treatment.

### Treatment with leptin resulted in increased PKC activity and sAPPα levels, an effect reversed upon treatment with chelerythrine and/or upon addition of AChE to A549 and H1299 cell media

PKC isoform expression and activation have been reported to be elevated in NSCLC compared to lung epithelial cells and linked to malignant progression of a variety of human cancers^43^. PKC signaling has been reported to regulate APP metabolism, increasing sAPPα release and decreasing Aβ secretion via both direct and indirect receptor-mediated PKC activation^29,44^. PKC signaling was found to be activated by muscarinic (M1/M3) ACh receptors increasing sAPPα release^29,45^. Blocking AChE activity was shown to increase PKC signaling and correlated with increased sAPPα release^29,46^. We have recently reported that the levels of sAPPα decreased in the media of A549 and H1299 cells upon treatment with the PKC inhibitor, chelerythrine^38^.

**Figure 5:**
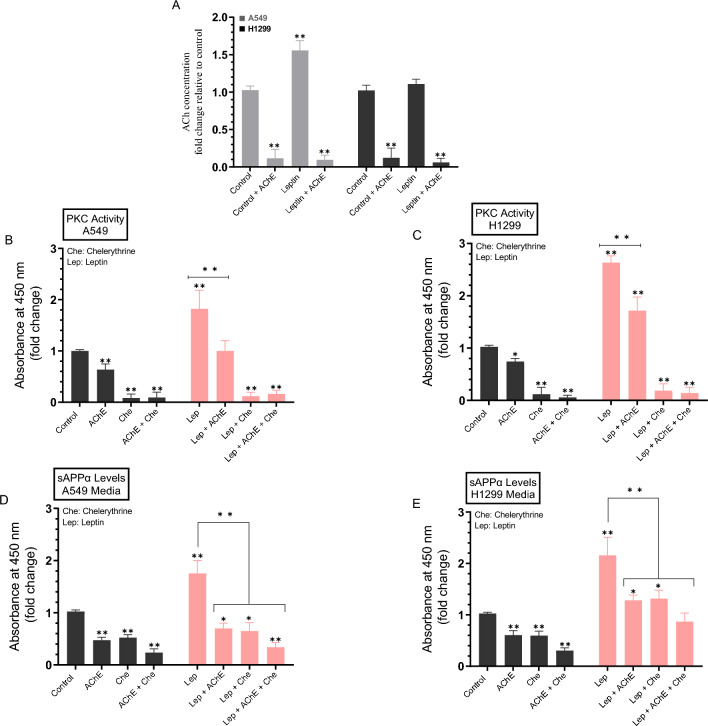
Leptin treatment led to increased PKC activity and the levels of sAPPα, an effect reversed upon treatment with chelerythrine and/or by addition of AChE to A549 and H1299 cell media. Cells (0.2 × 10^5^) were grown in 10% FBS-supplemented media for 24 h then serum-starved overnight. The media was then replaced with fresh serum-free media then the cells were either untreated or treated for 72 h with AChE (60 nM), the PKC inhibitor (chelerythrine, 7.5 μM), leptin (50 ng/mL), or in combination. The levels of ACh (**A**) in the cell media, the PKC activity in A549 (**B**) and H1299 (**C**) cells, the levels of sAPPα released into the culture media of A549 (**D**) and H1299 (**E**) cells during the 3-day incubation period were measured as described in the “Methods” section. Data from five independent assays, each carried out in triplicate, were averaged, normalized, and expressed as fold change relative to untreated cells (control) using the GraphPad 10.0.2 software. Asterisks indicate a statistically significant difference from the corresponding control of each cell line, Mann–Whitney test. Statistical differences between different groups were analyzed by an ordinary one-way analysis of variance (ANOVA) followed by Tukey’s post-hoc multiple comparison test. *p < 0.05, **p < 0.01. Absence of asterisks indicates no significance.

To examine the effect of leptin on the PKC activity and the levels of sAPPα, A549 and H1299 cells were grown in 10% FBS-supplemented media for 24 h then serum-starved overnight. The media was then replaced with fresh serum-free media then the cells were treated for 72 h with AChE, the PKC inhibitor (chelerythrine), leptin, or in combination (Fig. 5). The levels of ACh in the cell media, the PKC activity, and sAPPα levels released into the culture media during the 3-day incubation period were measured as described in the “Methods” section (Fig. 5).

To test the effects of ACh in the media on activation of PKC or the levels of sAPPα, we attempted to decrease ACh levels by addition of exogenous AChE (Fig. 5A). Exogenously added AChE was effective at decreasing the ACh levels in the media of A549 and H1299 cells with or without leptin treatment (Fig. 5A).

Treatment with AChE decreased the activity of PKC ~ 1.55-fold in A549 cells (Fig. 5B) and ~ 1.35-fold in H1299 cells (Fig. 5C). Leptin treatment of A549 cells led to ~ 1.80-fold increase in the PKC activity that was decreased by A549 cell co-treatment with leptin and AChE (Fig. 5B). Similar trends were observed when using H1299 cells with leptin treatment increasing the PKC activity ~ 2.65-fold, an effect that decreased ~ 1.50-fold upon H1299 cell co-treatment with leptin and AChE (Fig. 5C).

Consistent with our previous findings^38^, addition of AChE resulted in decreased sAPPα levels ~ 2.15-fold in A549 cell media (Fig. 5D) and ~ 1.65-fold in the media of H1299 cells (Fig. 5E) suggesting a role of AChE in shifting APP processing toward the amyloidogenic pathway and away from the non-amyloidogenic pathway. Blocking PKC activation with chelerythrine also decreased the levels of sAPPα ~ 1.95-fold in the media of A549 cells (Fig. 5D) and ~ 1.70-fold in H1299 cell media (Fig. 5E). Co-treatment of cells with AChE and chelerythrine (Fig. 5D,E) led to further decrease of sAPPα levels in the media (~ 4.20-fold decrease in A549 cell media and ~ 3.35-fold decrease in H1299 cell media). The levels of sAPPα increased in A549 cell media upon treatment with leptin ~ 1.70-fold (Fig. 5D) and ~ 2.00-fold in the media of H1299 cells (Fig. 5E). Compared with A549 cells treated with only leptin, the levels of sAPPα in the media were ~ 2.50-fold lower in cells co-treated with leptin and AChE, ~ 2.70-fold lower in cells co-treated with leptin and chelerythrine, and 5.15-fold lower in cells co-treated with leptin, AChE, and chelerythrine (Fig. 5D). Similarly, relative to H1299 cells treated with only leptin, the levels of sAPPα in the media were ~ 1.68-fold lower in cells co-treated with leptin and AChE, ~ 1.63-fold lower in cells co-treated with leptin and chelerythrine, and 2.50-fold lower in cells co-treated with leptin, AChE, and chelerythrine (Fig. 5E).

### Treatment with leptin resulted in increased ERK1/2 activity and sAPPα levels, an effect reversed upon treatment with AChE and/or PD98059, or in combination with chelerythrine

**Figure 6:**
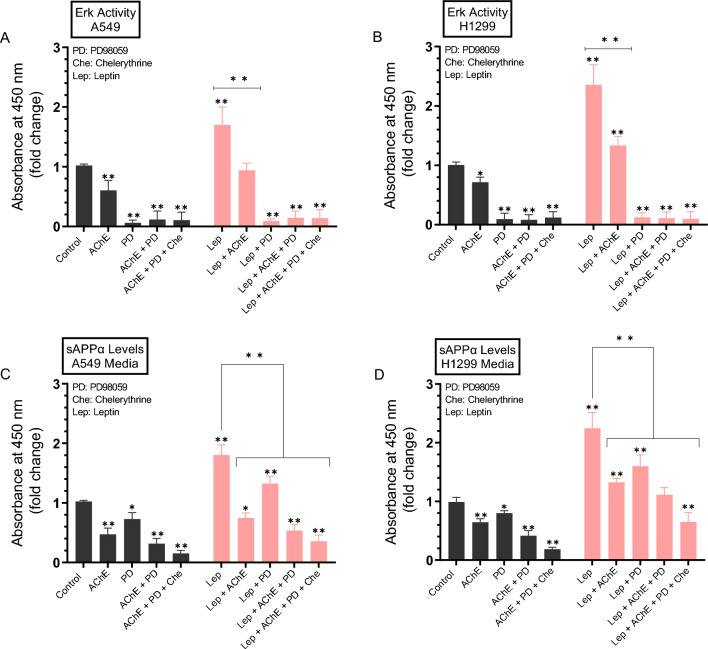
Cell treatment with AChE and/or PD98059 or in combination with chelerythrine, reversed the observed leptin-induced increase in ERK1/2 activity and sAPPα levels. Cells (0.2 × 10^5^) were grown in 10% FBS-supplemented media for 24 h then serum-starved overnight. The media was replaced with fresh serum-free media, then the cells were treated for 72 h with AChE (60 nM), the ERK1/2 inhibitor (PD98059, 50 μM), the PKC inhibitor (chelerythrine, 7.5 μM), leptin (50 ng/mL), or in combination. ERK1/2 activity was measured in A549 (**A**) and H1299 (**B**) cells as described in the “Methods” section. Levels of sAPPα released into the culture media of A549 (**C**) and H1299 (**D**) cells during the 3-day incubation period were measured as described in the “Methods” section on the same amount of protein (3 µL of 600 µg/mL total protein) of each sample. Data from five independent assays, each carried out in triplicate, were averaged, normalized, and expressed as fold change relative to untreated cells (control) using the GraphPad 10.0.2 software. Asterisks indicate a statistically significant difference from the corresponding control of each cell line, Mann–Whitney test. Statistical differences between different groups were analyzed by an ordinary one-way analysis of variance (ANOVA) followed by Tukey’s post-hoc multiple comparison test. *p < 0.05, **p < 0.01. Absence of asterisks indicates no significance.

Previously, we observed a more pronounced decrease in the levels of sAPPα in A549 and H1299 cell media upon blocking the activities of ERK1/2 and PKC with a combination of PD98059 and chelerythrine^38^. To examine the effect of leptin on the activity of ERK1/2 and sAPPα levels, cells were grown in 10% FBS-supplemented media for 24 h then serum-starved overnight. The media was replaced with fresh serum-free media then the cells were treated for 72 h with AChE, the ERK1/2 inhibitor (PD98059), the PKC inhibitor (chelerythrine), leptin, or in combination (Fig. 6).

ERK1/2 activity decreased ~ 1.65-fold upon treatment of A549 cells with AChE (Fig. 6A) and ~ 1.40-fold when H1299 cells were treated with AChE (Fig. 6B). Treatment with leptin resulted in ~ 1.70-fold increase in the activity of ERK1/2 in A549 cells (Fig. 6A) and ~ 2.40-fold increase in H1299 cells (Fig. 6B). Relative to leptin treatment alone, cell incubation with both leptin and AChE led to ~ 1.80-fold decrease in the activity of ERK1/2 in A549 cells (Fig. 6A) and ~ 1.75-fold decrease in the kinase activity in H1299 cells (Fig. 6B).

Relative to control, sAPPα levels decreased in the media of A549 cells ~ 2.15-fold with AChE treatment and ~ 1.35-fold upon cell incubation with PD98059 (Fig. 6C). Co-treatment of A549 cells with AChE and PD98059 decreased sAPPα levels ~ 3.15-fold while co-treatment of A549 cells with AChE, PD98059, and chelerythrine further decreased those levels ~ 6.65-fold (Fig. 6C). Similar results were obtained when using H1299 cells (Fig. 6D). Relative to control, sAPPα levels decreased in the media of H1299 cells ~ 1.55-fold with AChE treatment and ~ 1.25-fold upon cell incubation with PD98059 (Fig. 6D). Co-treatment of H1299 cells with AChE and PD98059 decreased sAPPα levels ~ 2.40-fold while co-treatment of H1299 cells with AChE, PD98059, and chelerythrine further decreased those levels ~ 5.40-fold (Fig. 6D).

Relative to A549 cell treatment with only leptin, sAPPα levels decreased in the media ~ 1.35-fold by co-treatment with leptin and PD98059, ~ 3.40-fold by co-treatment with leptin, AChE, and PD98059, and ~ 5.00-fold by co-treatment with leptin, AChE, PD98059, and chelerythrine (Fig. 6C). Relative to H1299 cell treatment with only leptin, sAPPα levels decreased in the media ~ 1.40-fold by co-treatment with leptin and PD98059, ~ 2.00-fold by co-treatment with leptin, AChE, and PD98059, and ~ 3.45-fold by co-treatment with leptin, AChE, PD98059, and chelerythrine (Fig. 6D).

### Treatment with leptin resulted in increased PI3K activity and sAPPα levels, an effect reversed upon treatment with AChE and/or LY294002, or in combination with chelerythrine or PD98059

**Figure 7:**
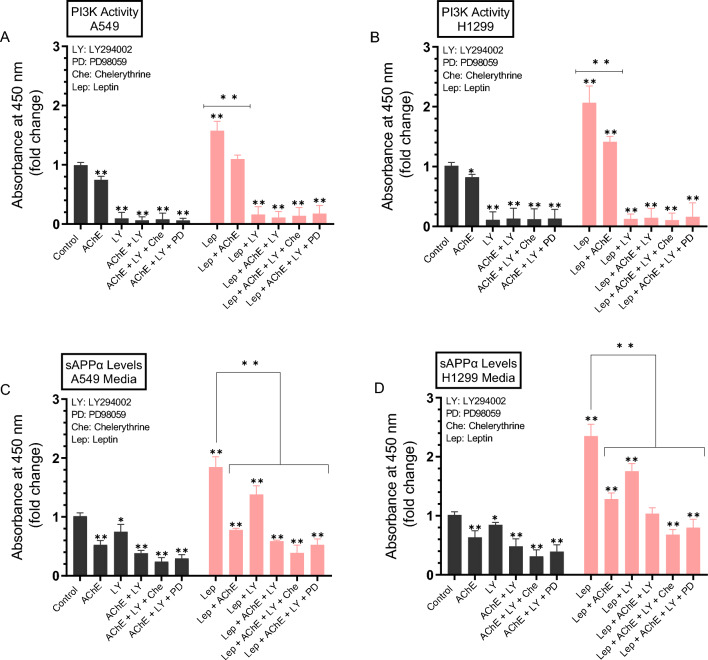
Treatment with AChE and/or LY294002, or in combination with chelerythrine or PD98059 reversed the leptin-induced increase in PI3K activity and sAPPα levels. Cells (0.2 × 10^5^) were grown in 10% FBS-supplemented media for 24 h then serum starved overnight. The media was then replaced with fresh serum-free media and the cells were then treated for 72 h with AChE (60 nM), the PI3K inhibitor (LY294002, 14.5 μM), the PKC inhibitor (chelerythrine, 7.5 μM), the ERK1/2 inhibitor (PD98059, 50 μM), leptin (50 ng/mL), or in combination. PI3K activity was measured in A549 (**A**) and H1299 (**B**) cells as described in the “Methods” section. Levels of sAPPα released into the culture media of A549 (**C**) and H1299 (**D**) cells during the 3-day incubation period were measured as described in the “Methods” section on the same amount of protein (3 µL of 600 µg/mL total protein) of each sample. Data from five independent assays, each carried out in triplicate, were averaged, normalized, and expressed as fold change relative to untreated cells (control) using the GraphPad 10.0.2 software. Asterisks indicate a statistically significant difference from the corresponding control of each cell line, Mann–Whitney test. Statistical differences between different groups were analyzed by an ordinary one-way analysis of variance (ANOVA) followed by Tukey’s post-hoc multiple comparison test. *p < 0.05, **p < 0.01. Absence of asterisks indicates no significance.

Previous findings showed that higher expression of leptin in NSCLC led to activation of PI3K/AKT signaling^42^. To examine whether leptin treatment affects the activity of PI3K and/or the levels of sAPPα under our conditions, cells were grown in 10% FBS-supplemented media for 24 h then serum starved overnight. The media was then replaced with fresh serum-free media and the cells were then treated for 72 h with AChE, the PI3K inhibitor (LY294002), the PKC inhibitor (chelerythrine), the ERK1/2 inhibitor (PD98059), leptin, or in combination (Fig. 7).

PI3K activity decreased ~ 1.35-fold upon treatment of A549 cells with AChE (Fig. 7A) and ~ 1.20-fold when H1299 cells were treated with AChE (Fig. 7B). The PI3K activity increased ~ 1.55-fold in A549 cells (Fig. 7A) and ~ 2.00-fold in H1299 cells (Fig. 7B) upon treatment with leptin. Relative to leptin treatment alone, cell incubation with both leptin and AChE led to ~ 1.45-fold decrease in the activity of PI3K in A549 and H1299 cells (Fig. 7A,B).

Relative to control, sAPPα levels decreased in the media of A549 cells ~ 1.90-fold with AChE treatment and ~ 1.35-fold upon cell incubation with LY294002 (Fig. 7C). Co-treatment of A549 cells with AChE and LY294002 decreased sAPPα levels ~ 2.65-fold. Co-treatment with AChE, LY294002, and chelerythrine further decreased sAPPα levels ~ 4.15-fold while co-treatment with AChE, LY294002, and PD98059 decreased sAPPα levels ~ 3.35-fold (Fig. 7C). Similar results were obtained when using H1299 cells (Fig. 7D). Relative to control, sAPPα levels decreased in the media of H1299 cells ~ 1.55-fold with AChE treatment and ~ 1.20-fold upon cell incubation with LY294002 (Fig. 7D). Co-treatment of H1299 cells with AChE and LY294002 decreased sAPPα levels ~ 2.00-fold (Fig. 7D). The levels of sAPPα decreased ~ 3.20-fold upon co-treatment of H1299 cells with AChE, LY294002, and chelerythrine and ~ 2.55-fold when cells were incubated with AChE, LY294002, and PD98059 (Fig. 7D).

Relative to A549 cell treatment with only leptin, sAPPα levels decreased in the media ~ 1.35-fold by co-treatment with leptin and LY294002, and ~ 3.15-fold by co-treatment with leptin, AChE, and LY294002 (Fig. 7C). The levels of sAPPα decreased ~ 4.75-fold upon co-treatment of A549 cells with leptin, AChE, LY294002, and chelerythrine and ~ 3.50-fold when cells were incubated with leptin, AChE, LY294002, and PD98059 (Fig. 7C) when compared to leptin only treatments. A comparable trend was observed when using H1299 cells (Fig. 7D). Relative to H1299 cell treatment with only leptin, sAPPα levels decreased in the media ~ 1.35-fold by co-treatment with leptin and LY294002, ~ 2.30-fold by co-treatment with leptin, AChE, and LY294002, ~ 3.45-fold upon co-treatment of H1299 cells with leptin, AChE, LY294002, and chelerythrine, and ~ 2.95-fold when cells were incubated with leptin, AChE, LY294002, and PD98059 (Fig. 7D).

### Cell viability increased by treatment of A549 and H1299 cells with leptin and decreased upon cell co-treatment with AChE, chelerythrine, PD98059, LY294002, or in combination

**Figure 8:**
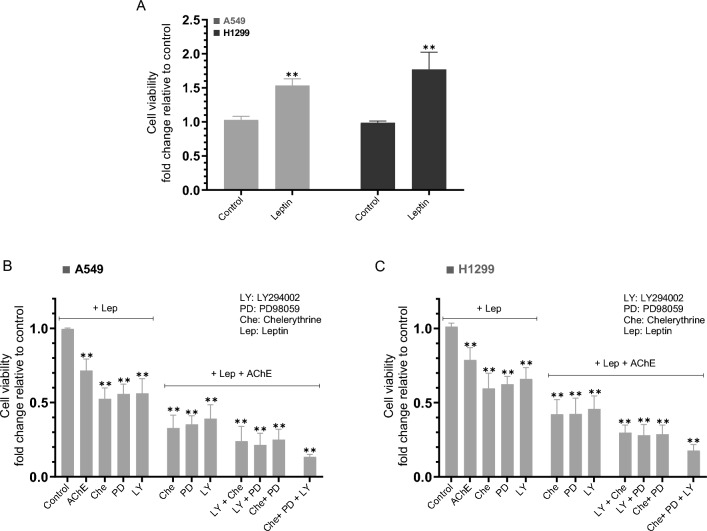
A549 and H1299 cell treatment with leptin enhanced cell viability, an effect reversed by cell treatment with leptin in the presence of AChE, chelerythrine, PD98059, LY294002, or in combination. Cells (0.2 × 10^5^) were grown in 10% FBS-supplemented media for 24 h. The following day, the cell monolayers were incubated in serum-free media overnight, then left either untreated for 72 h or treated with leptin (50 ng/mL) without or with the following co-treatments [AChE (60 nM), the PKC inhibitor (chelerythrine, 7.5 μM), the ERK1/2 inhibitor (PD98059, 50 μM), the PI3K inhibitor (LY294002, 14.5 μM)], or in combination. Cell viability with or without leptin (**A**) or with A549 co-treatments (**B**) or H1299 co-treatments (**C**) was measured as described in the “Methods” section. Data from five independent assays, each carried out in triplicate, were averaged, normalized, and expressed as fold change relative to untreated cells (control, **A**) or leptin controls (**B**,**C**) using the GraphPad 10.0.2 software. Asterisks indicate a statistically significant difference from the corresponding control of each cell line, Mann–Whitney test. **p < 0.01.

Leptin (50 ng/mL) was shown to act in an autocrine manner in A549 and H1299 cells to increase cell cycle progression promoting proliferation^42^. To examine the effect of leptin on A549 and H1299 cell viability, cells were grown in 10% FBS-supplemented media for 24 h. The following day, the cell monolayers were incubated in serum-free media overnight, then left either untreated for 72 h or treated with leptin (50 ng/mL) without or with AChE, the PI3K inhibitor (LY294002), the PKC inhibitor (chelerythrine), the ERK1/2 inhibitor (PD98059), or in combination (Fig. 8).

Cell treatment with leptin increased A549 cell viability ~ 1.55-fold and H1299 cell viability ~ 1.75-fold (Fig. 8A). Relative to A549 cells treated with only leptin, the fold decrease in cell viability was ~ 1.40-fold with AChE, and ~ 1.85-fold by treatment with either chelerythrine, PD98059, or LY294002 (Fig. 8B). A more pronounced fold decrease in viability was observed upon A549 cell co-treatment with leptin and AChE and single treatments with either chelerythrine, PD98059, or LY294002 (~ 3.00-fold), double treatments (~ 4.00-fold), or in the presence of the three inhibitors (~ 7.45-fold) (Fig. 8B). Similar trends were observed when using H1299 cells (Fig. 8C). Relative to H1299 cells treated with only leptin, the fold decrease in cell viability was ~ 1.25-fold with AChE, and ~ 1.60-fold by treatment with either chelerythrine, PD98059, or LY294002 (Fig. 8C). As observed when using A549 cells, a more pronounced fold decrease in viability was observed upon H1299 cell co-treatment with leptin and AChE and single treatments with either chelerythrine, PD98059, or LY294002 (~ 2.35-fold), double treatments (~ 3.35-fold), or in the presence of the three inhibitors (~ 5.60-fold) (Fig. 8C).

## Discussion

Leptin is widely recognized as a cytokine, pleiotropic hormone, and an obesity-associated adipokine that upon binding to its leptin receptor, regulates energy metabolism and appetite^12–14^. More recently, expression of both leptin and its receptor has been documented in cell types besides adipocytes that include cancer cells, suggesting a possible role in cancer signaling^12,15–18,21–23^. Both leptin and its receptor were found to be expressed in NSCLC at higher levels than in normal lung tissues^15,18–23,42,47^ and led to proliferation and antiapoptotic functions^19,42,47^. Expression levels of leptin were higher in four NSCLC cell lines, including A549 and H1299, than in normal control lung cells^42,48^. Higher expression levels of leptin were found in H1299 cells than in A549 cells^42^. Leptin was also shown to play an important role in increasing A549 cell growth and inhibiting apoptosis via blocking endoplasmic reticulum stress-mediated pathways^49^. Leptin was reported to play a role in upregulating TGF-β inducing epithelial–mesenchymal transition in A549 cells^48^.

Several reports have documented protective actions of leptin in several neurodegenerative models^12,14^. Expression of β- and γ-secretase, that sequentially cleave APP to generate toxic forms of Aβ, was found to be downregulated by leptin leading to enhanced cleavage of APP by α-secretase and decreased Aβ levels^12–14^. We have previously reported that the levels of intact Aβ40/42 were higher in A549 cell media than in the media of H1299 cells^35^ and correlated with higher sAPPα levels in H1299 cell media compared to the media of A549 cells^38^. Using different cell lines with the lack of a specific signaling component is an effective way to test pathway-specific hypotheses. Here, we used A549 (p53 wild-type) and H1299 (p53-null) with minimal levels of AChE in the media relative to the media of A549 cells to test the hypothesis that leptin regulates the amyloidogenic and non-amyloidogenic pathways via a mechanism, in part, involving p53 and AChE. In this study, we show that incubation with leptin increased the levels of sAPPα in the media of A549 and H1299 cells (Fig. 1) and that higher levels of sAPPα resulting from leptin treatment correlated with lower Aβ40/42 levels in the media (Fig. 2). Further support for the role of leptin in regulating the levels of sAPPα and Aβ40/42 was obtained by the finding that knockdown of leptin or its receptor led to reduced levels of sAPPα and increased levels of Aβ40/42 in the media of A549 and H1299 cells (Fig. 3).

Inhibition of p53 signaling was observed upon leptin expression in NSCLC cells suggesting a negative correlation between p53 and leptin signaling^42^. Our previously published work showed that IGFBP-3 inhibits hyaluronan-CD44 signaling via a mechanism that depends on both p53 and AChE and that the levels of AChE and activity were diminished in the media upon treatment of A549 cells, transfected with either p53 siRNA or with AChE siRNA, with IGFBP-3^36^. We also found that the levels of AChE are lower in the media of H1299 cells as compared to A549 cell media^36,38^. Based on our previous observations, we hypothesized that leptin negatively affects AChE. AChE is a member of the serine hydrolase family that is widely recognized for its classical role in the catalytic hydrolysis of cholinergic neurotransmitters^50^. Recent work has unraveled non-classical functions of the enzyme as a potential tumor growth suppresser^51^ and anticancer therapeutic^52,53^. Our results (Fig. 4) show that A549 cell treatment with leptin increased ACh levels and blocked the activities of AChE and p53 while opposite effects were found upon A549 cell transfection with siRNA targeting either leptin or its receptor.

Expression of PKC isoforms was previously reported to be higher in NSCLC as compared to lung epithelial cells^43^. Activation of PKC isoforms has been linked to proliferation and malignant progression of a number of human cancers^43^. PKC signaling has been previously shown as a central mechanism that regulates APP metabolism leading to enhanced sAPPα release and diminished Aβ secretion^29,44^. PKC signaling was reported earlier to be coupled to and activated by muscarinic (M1/M3) ACh receptors increasing release of sAPPα^29,45^. Inhibiting the activity of AChE led to increased PKC signaling and correlated with enhanced release of sAPPα^29,46^. In addition, previous reports have shown the participation of the MAPK-ERK pathway in the regulation of the α-secretase activity with the release of sAPPα inhibited by treatment with the ERK inhibitor, PD98059^29,46^.

Leptin has been reported to induce PKC and activate the Ras/ERK1/2 signaling cascade and the PI-3K/Akt pathway^16^. Recently, we published that the levels of sAPPα decreased in the media of A549 and H1299 cells upon treatment with the PKC inhibitor (chelerythrine), the ERK1/2 inhibitor (PD98059), or the PI3K inhibitor (LY294002)^38^. In this study, a phosphatase inhibitor cocktail was included in the lysis buffer used for kinase activity assays since kinases can be rapidly inactivated by dephosphorylation resulting in reduced kinase activity^54^. We found that treatment with leptin resulted in increased activity of PKC, ERK1/2, and PI3K and the levels of sAPPα, effects that were reversed by treatment with kinase inhibitors and/or upon addition of AChE to A549 and H1299 cell media (Figs. 5, 6, 7).

Previously, leptin was reported to function in an autocrine manner in A549 and H1299 cells promoting cell proliferation^42^. Our data (Fig. 8) support these observations showing that cell viability increased by treatment of A549 and H1299 cells with leptin and decreased upon co-treatment with AChE and/or PKC, ERK1/2, and PI3K inhibitors.

**Figure 9:**
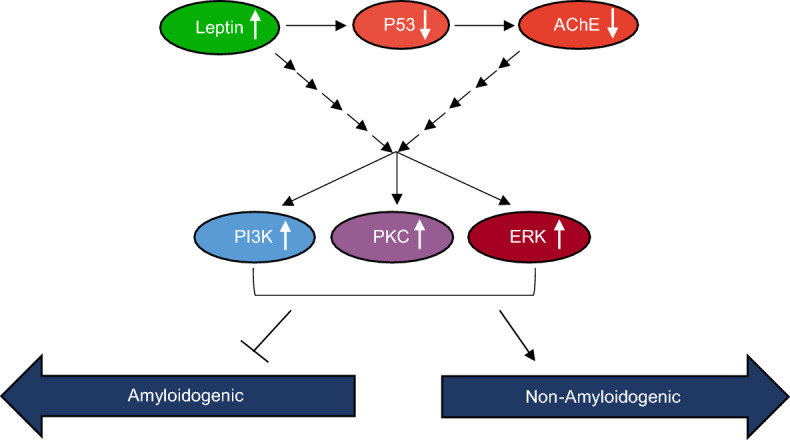
Schematic representation of the main hypothesis and findings from this study.

This study provides a new mechanism of sAPPα regulation in NSCLC cell media by leptin. In brief, leptin incubation led to (a) increased sAPPα levels in the media of A549 and H1299 cells that were accompanied by lower Aβ40/42 levels; (b) increased ACh levels and decreased activities of AChE and p53 in A549 cells; (c) enhanced activities of PKC, ERK1/2, and PI3K and sAPPα levels, effects that were reversed by treatment with kinase inhibitors and/or upon addition of AChE to A549 and H1299 cell media; (d) increased A549 and H1299 cell viability that was blocked by co-treatment with AChE and/or PKC, ERK1/2, and PI3K inhibitors. The cell lines used in this study provide several advantages as an in vitro model system for basic lung cancer research and anticancer drug design. However, one limitation of in vitro studies is that cells cannot mimic the complex environment of biological systems, representing a challenge in new drug development. Translating findings from this research of the molecular and cellular mechanisms employed by leptin in regulating the levels of sAPPα and NSCLC cell survival, to fully unravel the fundamental mechanisms operative in patient tumors, represents a limitation. Therefore, translational research targeting leptin and/or its receptor as therapeutic molecules and design of NSCLC animal experiments that mimic treatment regimens might be promising as therapeutic strategies for NSCLC cancer treatment, driving progress in the field of lung cancer research. Based on our results, we propose a model (Fig. 9) summarizing the main findings of this study.

## Methods

### Materials

Most of the material used in this study was purchased as we reported earlier^38,55–57^. Phosphate Buffered Saline (PBS), nitrocellulose membranes, streptavidin-horseradish peroxidase (HRP) conjugate, Ponceau S solution, LY294002 hydrochloride, hydrogen peroxide solution, human leptin (L4146), recombinant human AChE (C1682, UniProt accession ID: C9JD78), chelerythrine chloride, and PD98059 were purchased from Sigma-Aldrich. LEP Human siRNA (AM16708), leptin mouse monoclonal antibody (MA5-23740), α-tubulin mouse monoclonal antibody (DM1A), 3,3′,5,5′-tetramethylbenzidine (TMB), Halt Protease and Phosphatase Inhibitor Cocktail, BCA protein assay kit, SuperSignal West Pico luminol (chemiluminescence) reagent, and lipofectamine 2000 transfection reagent were from ThermoFisher. Donkey anti-mouse IgG (HRP) (ab205724), goat anti-AChE antibody (ab31276), and rabbit anti-Goat IgG H&L (HRP) (ab6741) were purchased from Abcam. Anti-Human Mouse sAPPα (2B3) IgG MoAb was purchased from IBL America. SignalSilence Control siRNA (Unconjugated, 6568) was purchased from Cell Signaling Technology. Anti-Aβ mouse (6E10, 1–16) antibody, anti-Aβ42 mouse antibody that is reactive to the C-terminus of Aβ42, anti-Aβ40 mouse antibody that is reactive to the C-terminus of Aβ40, and biotin anti-Aβ mouse (6E10, 1–16) antibody were from BioLegend. Ob-R siRNA (sc-36115) and Ob-R/leptin receptor antibody (sc-8391) were purchased from Santa Cruz.

### Cell culture

The human NSCLC cell lines, A549 (ATCC CCL-185) and H1299 (ATCC CRL-5803), were cultured as we reported earlier^31,32,35–37,58–61^ in 5 mL DMEM/F12 media/nutrient mixture supplemented with 10% Fetalgro bovine growth serum, 50 U/mL penicillin, and 50 U/mL streptomycin. Cells were allowed to grow in 25 cm^2^ tissue culture flasks overnight in an incubator at 37 °C, 5% CO_2_ and 95% humidity. Cells were counted, after trypan blue staining, using a hemocytometer. Cells were also treated with the protein kinase C (PKC) inhibitor (chelerythrine, 7.5 μM), mitogen-activated protein kinase kinase (MEK) inhibitor (PD98059, 50 μM), or phosphoinositide 3-kinase (PI3K) inhibitor (LY294002, 14.5 μM), where indicated.

### Quantitative determination of sAPPα

Using the quantitative sandwich enzyme immunoassay human sAPPα ELISA Kit (MyBioSource, MBS915453), the sAPPα concentration was measured in the cell culture supernatant, as we previously reported^38^ and according to the manufacturer’s instructions. Briefly, following incubation of wells pre-coated with an anti-sAPPα specific antibody with the samples, a biotin-conjugated antibody specific for sAPPα was added. The signal was then detected following incubation with avidin conjugated horseradish peroxidase (HRP) and a substrate solution.

### Quantitation of Aβ

Aβ ELISAs were carried out for determining the relative levels of Aβ according to previously published protocols^62–64^ and as we published earlier^31,35,38^. Briefly, two-site binding ELISAs were used to detect Aβ1–40 and Aβ1–42 (Aβ40/42). Anti-Aβ42 antibody that is reactive to the C-terminus of Aβ42, or anti-Aβ40 antibody that is reactive to the C-terminus of Aβ40 were used as the capture antibodies. Following incubation with the media, the wells were washed, then biotinylated-anti-Aβ 6E10 (to Aβ1–16) antibody was added as the detection antibody. The signal was quantitated following addition of streptavidin-horseradish peroxidase and the TMB substrate. Negative controls included binding conditioned media or pure Aβ peptides [Aβ40-HFIP (AS-64128-05), Aβ42-HFIP (AS-64129-05)] then adding all components along with streptavidin-horseradish peroxidase and TMB, but without addition of biotin-6E10 antibodies. To allow conversion of the OD measurements to concentrations of bound material, wells were coated with 2.5, 10, 50, 100, 500, and 5000 nM pure Aβ40 and Aβ42 peptides and probed with biotin-6E10 antibodies. The OD was corrected for non-specific binding by subtracting the mean background absorbance for the negative controls from all data points prior to analysis. All absorbance measurements were taken in the linear range. The same concentration of the samples from the same treatments were also added to ELISA wells and probed using only biotinylated-anti-Aβ 6E10 antibodies. Statistical analysis was determined by the GraphPad Prism 10.0.2 software.

### Western blotting

Cell extracts or lysate supernatants collected as indicated were analyzed according to protocols we published previously^36–38,56^. Briefly, attached live cells were harvested and the cell pellet was resuspended in lysis buffer, 1 mM PMSF, and Halt protease and phosphatase inhibitor cocktail. After a brief sonication, samples were centrifuged, and the supernatants were stored at − 80 °C until further analysis. Following methods we reported previously^36–38,56,58^, samples, with the same amount of protein measured using the BCA protein assay kit, were fractionated on 15% SDS-PAGE then transferred onto a nitrocellulose membrane. The membrane was blocked then incubated with the primary and secondary antibodies. Proteins were detected by using SuperSignal West Pico luminol (chemiluminescence) reagent, and the Western blot was imaged with a Bio-Rad molecular imager.

### Dot blotting

Total protein samples of the conditioned media, 3 µL of 600 µg/mL, obtained after the indicated cell treatments, were spotted onto a nitrocellulose membrane as we reported earlier^31,35,36,58^. The membrane was blocked using TBST containing 5% BSA for 1 h at RT, then incubated with goat anti-AChE antibodies overnight at RT. After washing 3× with TBST, the membrane was incubated with anti-goat IgG-HRP for 30 min at RT. After washing, the amount of AChE on the membrane was detected using SuperSignal West Pico luminol (chemiluminescence) reagent. The blots were imaged with a Bio-Rad molecular imager and quantitated using Image J 1.47 v software. Recombinant human AChE was used as a positive control and distilled water was used as a negative control.

### Leptin assay

The levels of leptin in the cell lysates were quantitated using the human leptin solid-phase sandwich ELISA (KAC2281, ThermoFisher). Briefly, samples were added to microplate wells pre-coated with a monoclonal antibody (capture) specific for human leptin. A second biotinylated monoclonal antibody (detector) was then added along with streptavidin-peroxidase. After washing, a substrate solution was added producing a colored product. The intensity of the colored product was directly proportional to the concentration of leptin in the sample.

### Leptin receptor assay

The human leptin receptor ELISA kit (ab282876) was used to quantitate the levels of the human leptin receptor protein in cell extracts following the manufacturer’s instructions. Briefly, the assay employs capture antibodies conjugated to an affinity tag that is detected by a monoclonal antibody used to coat the ELISA wells, allowing formation of the antibody-analyte sandwich complex in one step.

### Quantitation of ACh concentrations

The choline/acetylcholine assay kit (ab65345) was used to measure the concentration of ACh according to the manufacturer’s recommendation and as we reported earlier^38^. The amount of ACh in the samples was calculated by subtracting choline from total choline (choline + ACh).

### AChE activity

The activity of AChE in the conditioned media was measured using the AChE Activity Assay Kit (MAK119) according to our earlier methods^36^ and those previously reported^65,66^. The colorimetric product was proportional to the activity of AChE in the samples.

### p53 transcription factor activity assay

The human p53 transcription factor activity assay kit (TFEH-p53, RayBio) was used to assay the activity of p53 as we reported earlier^56,60,61^. In brief, active p53 present in whole cell lysates was specifically captured by double-stranded oligonucleotides containing the p53 binding sequence, bound to 96-well plates.

### PKC assay

The PKC kinase activity assay kit (Abcam, ab139437) was used as we previously reported^38^. In this ELISA, a polyclonal antibody was used to detect phosphorylation of a specific PKC synthetic peptide.

### ERK assay

ERK activity was quantitated using the ERK1/2 (pT202/Y204 + Total) ELISA kit (Abcam, ab176660) according to the manufacturer’s instructions and as we previously published^38,61^. Signals for phospho-ERK1/2 and total-ERK1/2 were normalized to cell number. The ratio of phospho-ERK1/2 to total-ERK1/2 for each treatment was then determined and plotted.

### PI3K assay

The PI3-kinase p85-alpha/gamma (Phospho-Tyr467/199) ELISA kit (Boster, EKC2337) was used following the instructions provided by the manufacturer and as we previously reported^56,60,61^. Primary antibodies targeted against total PI3K p85 and phosphorylated-PI3K p85 (recognizes p85 PI3K alpha/gamma phospho-tyrosine 467/199) were used. The signals for phospho-PI3K and total-PI3K were each normalized to cell number, determined using a crystal violet solution. The ratio of phospho-PI3K to total-PI3K was then calculated and plotted for each treatment.

### MTT assay

Cell viability was measured using the MTT reduction assay (Sigma-Aldrich) as we reported previously^36,58,67^. All absorbance measurements (570 nm) were in the linear range. As control, untreated cells (positive control) or wells containing only cell-free media (negative control) were used.

### SiRNA transfection

Transfections were carried out according to our previous methods^36–38,56^. Control siRNA and leptin siRNA were each mixed with Lipofectamine 2000 transfection reagent diluted in Opti-MEM Media (ThermoFisher) according to instructions provided by the manufacturer. The mixtures were then added to cells at a final concentration of 100 nM for each siRNA. The cells were allowed to incubate at 37 °C for 12 h followed by the specific indicated treatments. Each measurement represents the mean ± SD of three–five independent experiments, each carried out in triplicate.

### Statistical analysis

The analysis was carried out as we reported earlier^31,35,36,59^*.* To evaluate the statistical differences, the Mann–Whitney or an ordinary one-way ANOVA followed by Tukey’s post-hoc multiple comparison test was performed. GraphPad Prism (GraphPad Software, 10.0.2) was used for statistical analysis.

### Supplementary Information

Supplementary Figures.

## Data Availability

All data generated or analyzed during this study are included in this article.
