# Supplementary material for: The role of leptin in regulation of the soluble amyloid precursor protein α (sAPPα) levels in lung cancer cell media
Source: Sci Rep. 2024 Feb 28;14:4921. doi: 10.1038/s41598-024-55717-y (PMC10901813; doi:10.1038/s41598-024-55717-y)

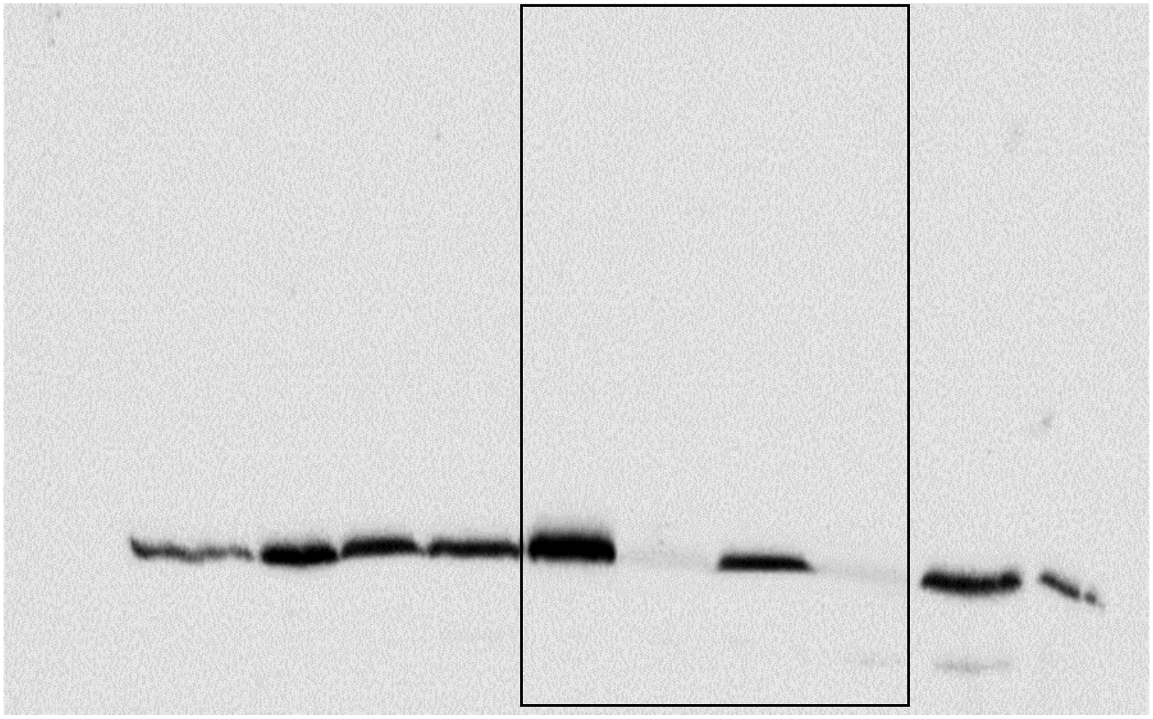

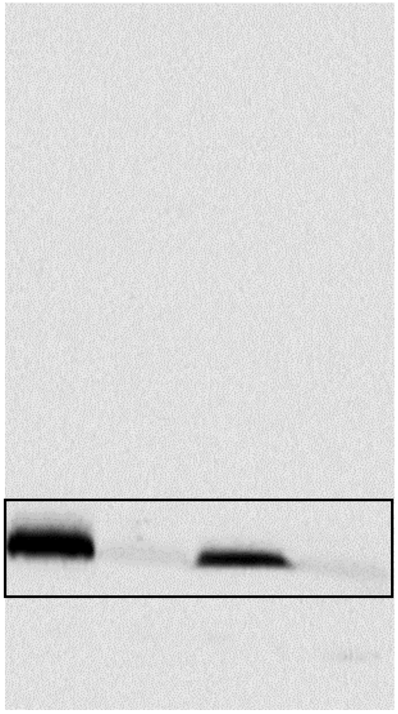

The image above was cropped to produce the image on the left.

The image on the left was cropped to produce the image below.

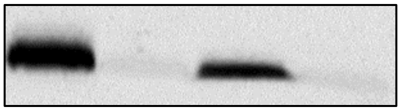

Shown on Figure 3A Top

The original blot above (Top) was cut to include and focus on the leptin samples above (Figure 3A top). This cut blot was stripped using Restore Western Blot Stripping Buffer (ThermoFisher) according to instructions provided by the manufacturer and re-probed using the antibody below (Figure 3A bottom). This was, in part, done to concentrate the antibodies on the more narrow area of the membrane.

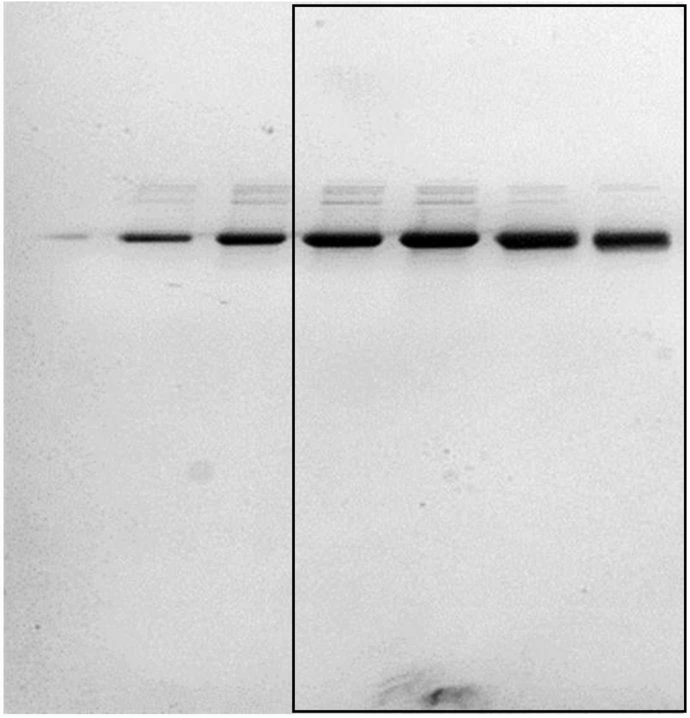

The image above was cropped to produce the image on the right.

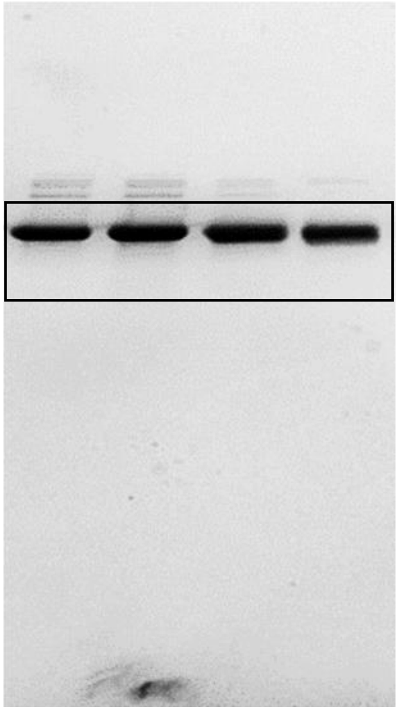

The image above was cropped to produce the image below.

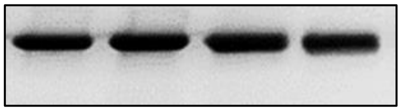

Shown on Figure 3A Bottom

Quantitation of Figure 3A

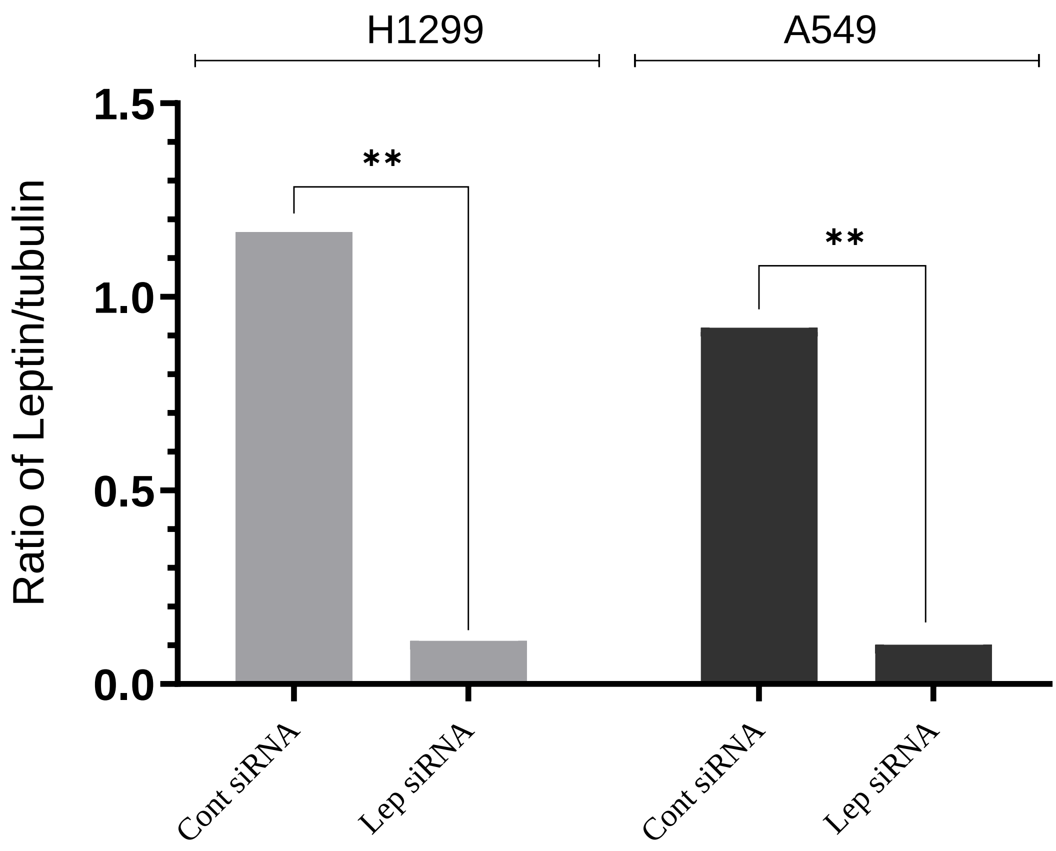

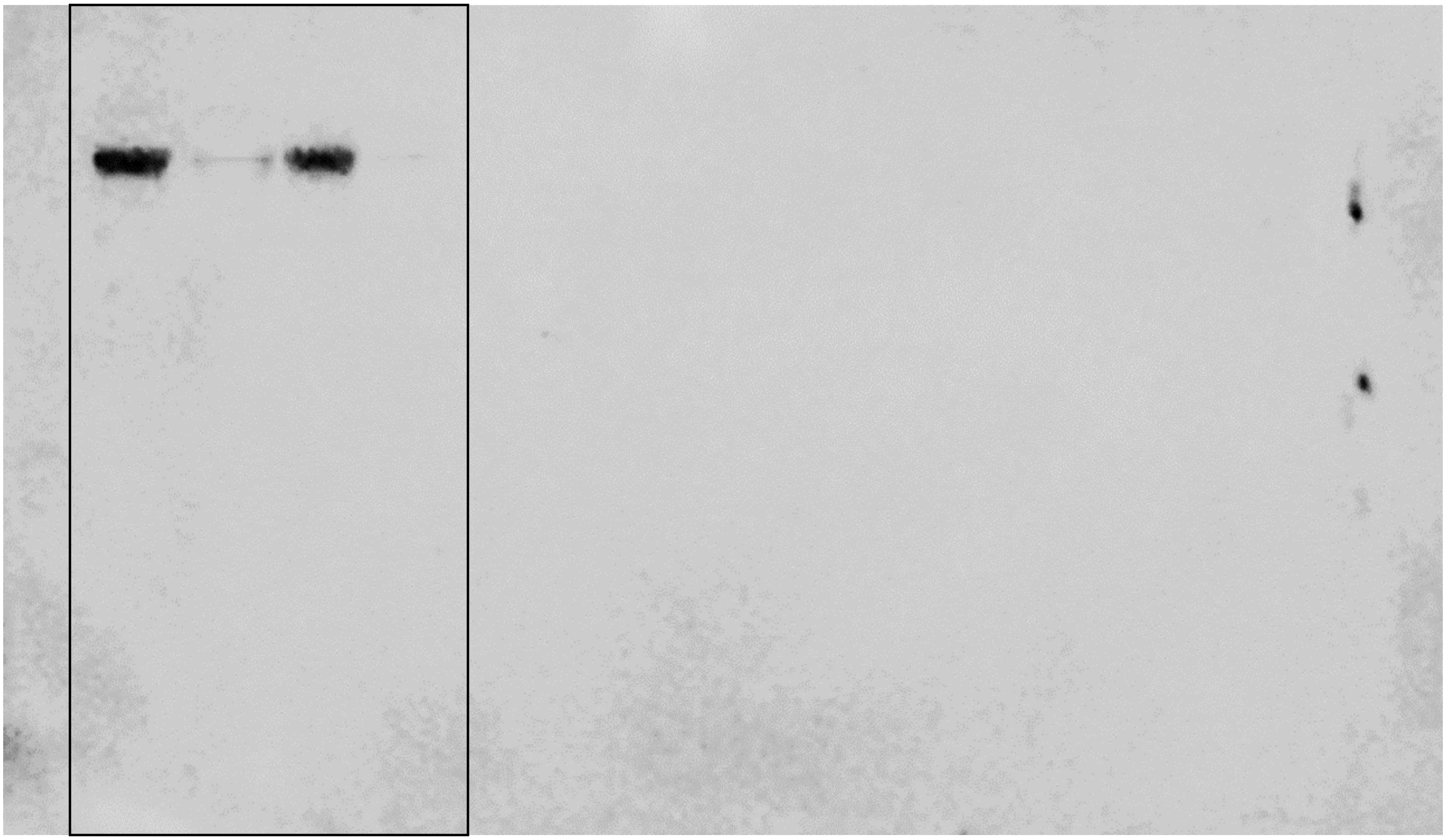

The image above was cropped to produce the image below

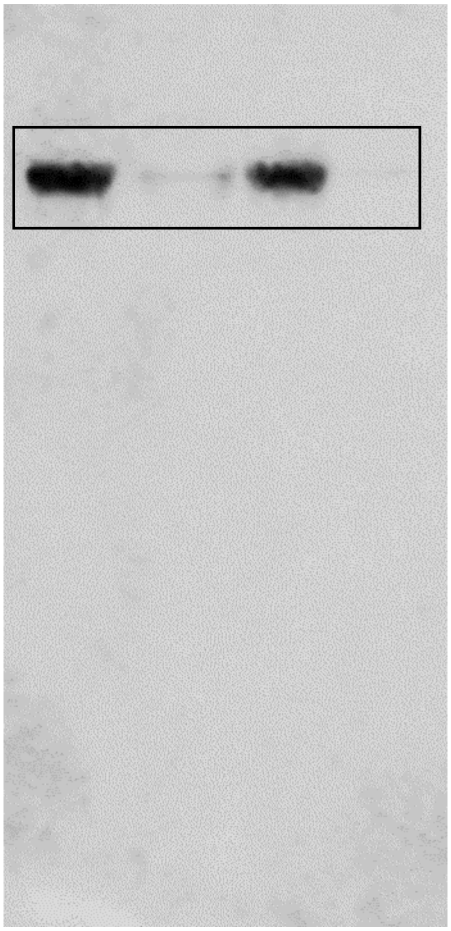

The image above was cropped to produce the image below.

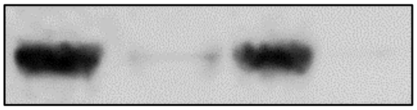

Shown on Figure 3B Top

The original blot above (Figure 3B top) was cut to include and focus on the leptin receptor samples above. This cut blot was stripped using Restore Western Blot Stripping Buffer (ThermoFisher) according to instructions provided by the manufacturer and re-probed using the antibody below (Figure 3B bottom). This was, in part, done to concentrate the antibodies on the more narrow area of the membrane.

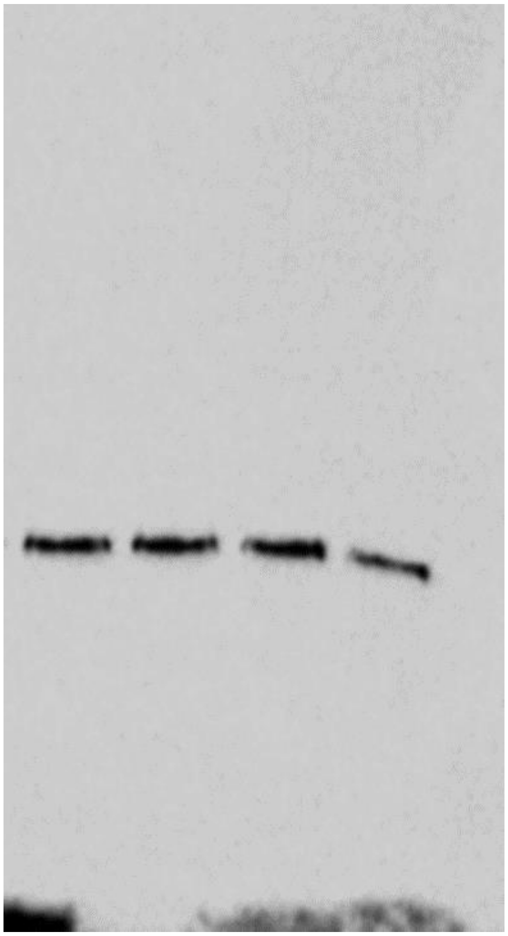

The image on the left was cropped to produce the image on the right.

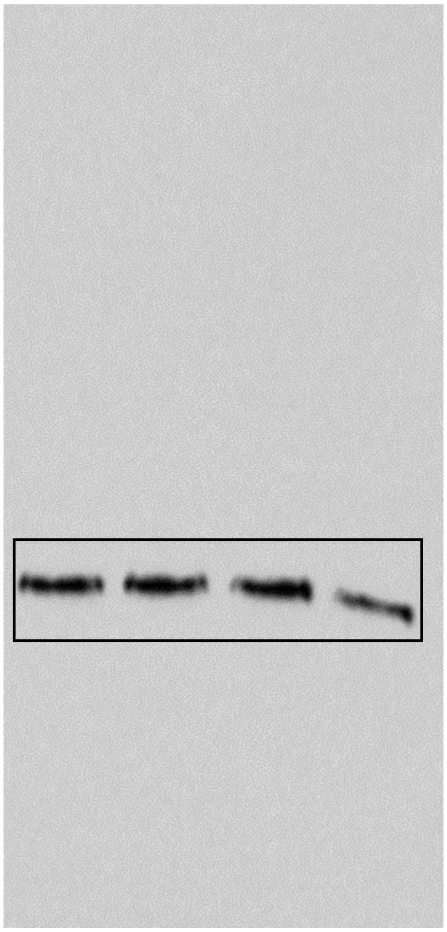

The image above was cropped to produce the image below.

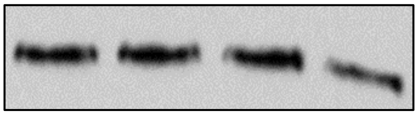

Shown on Figure 3B Bottom

Quantitation of Figure 3B

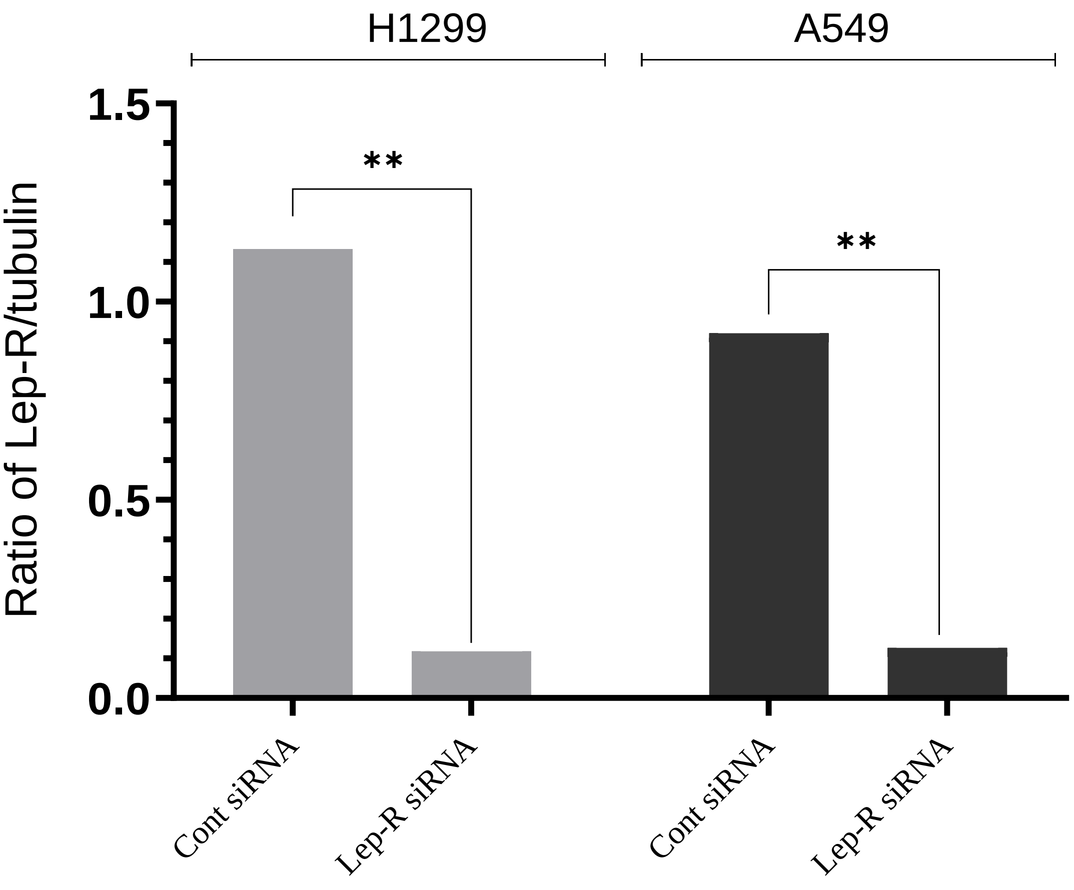

Supplement: Supplementary file 1 — Supplementary Figures. [file 41598_2024_55717_MOESM1_ESM.pdf]
